# Activated Scavenger Receptor A Promotes Glial Internalization of Aβ

**DOI:** 10.1371/journal.pone.0094197

**Published:** 2014-04-09

**Authors:** He Zhang, Ya-jing Su, Wei-wei Zhou, Shao-wei Wang, Peng-xin Xu, Xiao-lin Yu, Rui-tian Liu

**Affiliations:** 1 National Key Laboratory of Biochemical Engineering, Institute of Process Engineering, Chinese Academy of Sciences, Beijing, China; 2 Tsinghua University School of Medicine, Haidian District, Beijing, China; 3 School of Life Science, Ningxia University, Yinchuan, China; Massachusetts General Hospital and Harvard Medical School, United States of America

## Abstract

Beta-amyloid (Aβ) aggregates have a pivotal role in pathological processing of Alzheimer’s disease (AD). The clearance of Aβ monomer or aggregates is a causal strategy for AD treatment. Microglia and astrocytes are the main macrophages that exert critical neuroprotective roles in the brain. They may effectively clear the toxic accumulation of Aβ at the initial stage of AD, however, their functions are attenuated because of glial overactivation. In this study, we first showed that heptapeptide XD4 activates the class A scavenger receptor (SR-A) on the glia by increasing the binding of Aβ to SR-A, thereby promoting glial phagocytosis of Aβ oligomer in microglia and astrocytes and triggering intracellular mitogen-activated protein kinase (MAPK) signaling cascades. Moreover, XD4 enhances the internalization of Aβ monomers to microglia and astrocytes through macropinocytosis or SR-A-mediated phagocytosis. Furthermore, XD4 significantly inhibits Aβ oligomer-induced cytotoxicity to glial cells and decreases the production of proinflammatory cytokines, such as TNF-α and IL-1β, in vitro and in vivo. Our findings may provide a novel strategy for AD treatment by activating SR-A.

## Introduction

Alzheimer’s disease (AD), the most prominent cause of senile dementia, is characterized by two major neuropathological hallmarks as extracellular deposits of fibrillar beta-amyloid (Aβ) and intracellular neurofibrillary tangles composed of hyperphosphorylated tau [1]. Aβ peptides are generated through sequential proteolytic processing of the amyloid precursor protein. Overproduction or insufficient clearance of Aβ breaks Aβ homeostasis and leads to overall elevation of its steady-state levels, resulting in Aβ aggregation and deposition, which contribute to AD pathogenesis [2]. Soluble Aβ oligomers rather than monomers or fibrils are highly neurotoxic species that may trigger the synapse loss and neuronal damage prior to the formation of senile plaques in the early stages of AD [3]. Aβ oligomers may induce membrane-related toxicity by perturbation of the membrane fluidity or structure, release of lipids from the neuronal plasma membrane, or formation of ion channels [4]. Moreover, Aβ aggregates may induce the production of reactive oxygen species (ROS), cytokines, and chemokines, such as TNF-α, IL-1β, IL-6, MCP-1, IL-8 and NO, leading to an initial neurodegenerative characteristic of AD [5]. Therefore, inhibiting Aβ production or promoting Aβ clearance is a rational strategy for the prevention or treatment of AD.

Several methods can be used to clear Aβ from the brain, such as degrading enzymes, LRP receptor or phagocytosis. Glial phagocytosis and degradation of Aβ are believed to be key mechanisms of the initial defense of the brain against the toxic accumulation of Aβ. With the advancement of AD pathology, microglia are overactivated and lose their intrinsic beneficial function of uptake and degradation of Aβ. These phenomena result in Aβ accumulation, release of ROS, cytokines, and chemokines, as well as chronic neuroinflammation, and thus, neurodegeneration in AD [6]. Therefore, modulating microglia activation may be a powerful strategy to clear Aβ and prevent Aβ-associated pathological events and neuronal loss.

Previous studies have demonstrated that microglia take up and degrade soluble and fibrillar Aβ in different ways. Soluble Aβ is internalized into microglia through constitutive, nonsaturable, and fluid phase macropinocytosis, and the internalized soluble Aβ is rapidly delivered to the lysosomes by the late endolytic pathway [7]. Fibrillar Aβ interacts with the cell surface innate immune receptor complex, initiates intracellular signaling cascades, and then stimulates phagocytosis [8], [9]. Multiple cell surface receptors, including class A scavenger receptor (SR-A), class B scavenger receptor type I (SR-BI), CD36, α6β1 integrin, CD14, CD47 and toll-like receptors (TLRs), reportedly mediate Aβ uptake and degradation by microglia [10]–[13].

The roles of members of SRs in AD are diverse, but SR-A and CD36 are the main receptors responsible for Aβ clearance. SR-A-mediated interactions with Aβ promote Aβ phagocytosis and clearance and downregulate inflammatory gene expression in dendritic cells by suppressing TLR4-induced activation of the transcription factor NF-κB [14]. Meanwhile, the interactions of CD36 with Aβ initiate a tyrosine kinase-based signaling cascade, induce the production of neurotoxins and proinflammatory molecules, and promote the recruitment of microglia to Aβ deposits in the brain [15], [16]. The activation of SR-A can induce ligand internalization and direct the traffic of the endocytic signaling transaction pathways, such as PI3K/NFκB and mitogen-activated protein kinases (MAPKs)/JNK/p38 [17], [18]. Moreover, astrocytes, the main glial cells in the brain, can also engulf and degrade Aβ, which is mediated by the receptors, such as CD36, CD47 and RAGE [19], [20]. Recently, a study on the SR-A-deficient AD transgenic mouse model showed that SR-A deficiency markedly accelerated Aβ accumulation and led to increased mortality, while pharmacological upregulation of SR-A expression increased Aβ clearance [21]. Therefore, the activation of some glial receptors such as SR-A may be beneficial for Aβ clearance and AD treatment.

## Materials and Methods

### Materials

Aβ_1–42_ was purchased from American Peptide Company (Sunnyvale, CA, USA). XD4 was synthesized by GL Biochem Co., Ltd. (Shanghai, China). The Aβ_1–42_ and oAβ_1–42_ kits for Aβ measurement were purchased from IBL Co., Ltd. (Gunma, Japan). Anti-Aβ antibody 6E10 (monoclonal raised against Aβ N-terminal) and 4G8 (monoclonal raised against Aβ17-24) were purchased from Signet Laboratories (Covance, Dedham, MA, USA). Antibodies against SR-A and CD36 were purchased from AbD Serotec (Oxford, UK) and Abcam (Cambridge, UK), respectively. Rabbit anti-His-tag polyclonal antibody was from 4bio Co. (Beijing, China). Fucoidan was obtained from Sigma–Aldrich (St. Louis, MO, USA). Poly-L-lysine was obtained from Sciencell (CA, USA). Transmembrane protein extraction kit was purchased from Merck Millipore (MA, USA). Cell-counting kit (CCK)-8 was purchased from Dojindo (Kumamoto, Japan). Phosphotracer ELISA kit was purchased from Abcam (Cambridge, UK).

### Monomer Aβ_1–42_ (mAβ_1–42_) and Oligomer Aβ_1–42_ (oAβ_1–42_) Preparation

Aβ_1–42_ was dissolved in 100% 1,1,1,3,3,3-hexafluoro-2-propanal (HFIP) to a concentration of 1 mg/mL, sonicated in a water bath for 10 min, aliquoted into microcentrifuge tubes, dried under vacuum, and stored at −20°C. To prepare the Aβ_1–42_ monomer, aliquoted Aβ_1–42_ films were reconstituted by dimethyl sulfoxide (DMSO) at a concentration of 1 mg/ml, sonicated for 1 min, and immediately added to the serum-free cell media. oAβ_1–42_ was prepared as previously described, with a slight modification [22]. Briefly, the stored peptide film was resuspended in DMSO to a concentration of 5 mM and diluted by the cell media or PBS to a final concentration of 100 μM. The media was then rotated at 4°C for 24 h to form the oligomer, and the oligomer–containing media was centrifuged at 14,000 g at 4°C for 10 min to remove the fibrillar and insoluble aggregated Aβ_1–42_. Prior to incubation with XD4, the medium was filtered through 0.22 μm filters (Millipore, MA, USA) to eliminate the residual fibrillar Aβ_1–42_.

### Cell Culture

Human microglia and rat astrocytes were purchased from ScienCell Research Laboratories (San Diego, CA, USA). They were isolated from human brain tissue and from day 2 rat cerebral cortex, respectively. Cells were delivered frozen at secondary culture. Upon initiating the sub-culture from cryopreserved cells, the astrocyte medium (ScienCell, No. 1801) and microglia medium (Sciencell, No. 1901) was used. Cells were passaged upon reaching 90% confluence to new poly-L-lysine coated plates, the media was changed every other day before 50% confluence or every day until 90% confluence. The cells used for Aβ_1–42_ uptake assay were at passage numbers 2 to 5. The immortalized murine BV-2 microglial cells were maintained in Dulbecco’s modified Eagle medium (DMEM) supplemented with 10% FBS (Hyclone, USA) and 1% penicillin–streptomycin (Hyclone, USA). Mouse primary microglia was provided by Pricell Corp. (Wuhan, China). The cells were derived from postnatal day 0–2 pups and isolated from mixed cultures at 14 days in vitro. Upon receipt, primary microglia was directly seeded onto a poly-L-lysine pre-coated six-well plate and cultured overnight with the microglia medium provided by the manufacturer (Pricell, MED-0014). Human astroglioma (U251) cells were maintained in low-glucose DMEM (Hyclone, USA) supplemented with 10% FBS and 1% penicillin–streptomycin (Hyclone, USA), and then seeded at a six-well-plate at passage numbers 5 to 6. All the cells were kept at 37°C in 5% CO_2_ atmosphere.

### Intracellular Aβ_1–42_ Quantification

Cells were pretreated with or without fucoidan at the indicated concentrations for 20 min, washed twice by PBS, followed by incubation with 1 μg/ml mAβ_1–42_ for 3 h or 1 μM oAβ_1–42_ for 1.5 h in the presence or absence of the indicated concentration of XD4. For antibody blockade assays, antibodies against SR-A (10 μg/ml) or CD36 (10 μg/ml) were incubated with BV-2 microglia for 30 min in serum-free media, and then 1 μM oAβ_1–42_ or preincubated oAβ_1–42_ with XD4 was added to the cells and incubated for 1.5 h. The cells were washed extensively with PBS and digested with trypsin (Hyclone, USA) for 3 min. Then the cells were lysed with WIP lysis buffer (Bioss Co., China). The Aβ_1–42_ levels in the lysates were quantified by ELISA using the Aβ_1–42_ immunoassay kits. Protein amounts in each experimental group were determined using a bicinchoninic acid protein assay (BCA) kit (Pierce, Rockford, IL, USA). Both mAβ_1–42_ and oAβ_1–42_ levels internalized by cells were standardized to cell protein concentration and normalized as a percentage of control (treatment of Aβ_1–42_ alone).

### Immunocytochemistry

A confocal microscope was used to localize Aβ_1–42_ and XD4 in BV-2 cells. Cells were seeded into a 12-well plate at a concentration of 5×10^6^ per well on coverslips in phenol red-free DMEM (Hyclone, USA) and incubated for 1 d. The cells were treated with 0.25 μM mAβ_1–42_ or mAβ_1–42_ mixed with His-XD4 (molar ratio 1∶50 and 1∶100) in serum-free media for 1.5 h. After washing twice with PBS, cells were fixed with 4% paraformaldehyde at 37°C, washed by PBS and blocked with 5% goat serum in PBS at room temperature for 2 h, and then incubated with primary anti-Aβ_1–42_ antibody 4G8 (1∶500) overnight at 4°C. Consequently, the cells were washed five times with PBS, incubated with goat-anti mouse FITC (1∶1000) or rabbit-anti his TIRTC (1∶1000) secondary antibody simultaneously in 10% goat serum for 1.5 h, and then incubated with 4′,6-diamidino-2-phenylindole (DAPI) in anti-fade mounting medium (Beyotime, Beijing, China) at room temperature for 2 h away from light. The coverslips were kept at 4°C until examination by a confocal laser scanning microscope (Zeiss LSM780). The control experiments were performed according to the same protocol without primary antibodies against Aβ_1–42_ and His-XD4.

### Membrane Protein Extraction

Membrane protein of BV-2 cells was prepared by trans-membrane protein extraction kit (Merck Millipore, MA, USA) according to the manufacturer’s instruction. Briefly, 1×10^7^ BV-2 cells were collected with a cell scraper and treated with a cytosolic extraction buffer. After centrifugation, the supernatants were collected as cytosolic (soluble) protein fraction. The remaining pellets were resuspended with detergent-free membrane protein extraction buffer with the addition of 5 μl protein inhibitor cocktail. After incubation and centrifugation, the supernatants enriched with integral membrane proteins were collected in a fresh tube, and the total protein concentration of the cytosolic and the membrane fraction were calculated by BCA assay kit.

### Immunoprecipitation

Approximately 30 μg of 6E10 antibody was cross-linked to 50 μl slurry of aminolink coupling resin with sodium cyanoborohydride according to the instructions of the manufacturer. The antibody-coupled resins were incubated with oAβ_1–42_, which were pre-incubated with or without XD4 for 2 h and added to homogenized membrane extracts of BV-2 cells. The mixture was incubated for 4 h at room temperature with gentle agitation at 30 min intervals. After the resin was washed four times with PBS, the immune-complex was collected in elution buffer and subjected to subsequent western blot procedures. The control experiment was performed with the same procedure in the absence of oAβ_1–42_.

### Western Blot

To identify the abundance of SR-A receptor in the immune-complexes generated in immunoprecipitation, the sample was resolved on 12% SDS-PAGE and transferred onto nitrocellulose membranes. The membranes were washed, blocked by 5% non-fat dried milk for 1 h at room temperature, and then incubated with rat-anti SR-A primary antibody (1∶1000) overnight at 4°C. The membranes were washed three times in 1% PBST and incubated with mouse anti-rat HRP-conjugated secondary antibody (1∶3000) at room temperature for 2 h. The blots were washed with PBS before being exposed to ECL reagent (Beyotime, Beijing, China).

### Cell Viability Assay

Colorimetric CCK-8 assay was used to determine the effect of XD4 on oAβ_1–42_-induced cytotoxicity on glia cells. Glia cells were seeded in 96-well plates at a density of 1×10^4^ per well. oAβ_1–42_ was pre-incubated with or without different concentrations of XD4 for 1 h before adding to the cells. The final concentration of oAβ_1–42_ in each culture well was 5 μM, and the molar ratios of oAβ_1–42_ to XD4 were 1∶2 and 1∶10, respectively. The plates were then incubated for 48 h at 37°C. Cell viability was monitored by adding 10 μl of CCK-8 solution reagent to each well and incubated at 37°C for 2 h. The absorbance of the wells was read at 450 nm using an MD SpectraMax M5 microplate reader. Averages from three replicate wells were used for each sample, and each experiment was repeated three times. Cell viability was calculated by dividing the absorbance of the wells containing samples by the absorbance of the wells containing medium alone.

### Kinase Phosphorylation Assay

To investigate the effect of XD4 on SR-related protein phosphorylation within the MAPK signaling pathway, the relative amount of phosphorylated p38 MAPK and JNK 1/2/3 was determined by Phosphotracer ELISA kits (Abcam, ab119674) in a semi-quantitative method. Cells were seeded at 96-well plates, and then 1 μM oAβ_1–42_ was mixed with or without XD4 (molar ratio 1∶25) added to the cell culture. The amount of the phosphorylated protein was determined by the fluorescence intensity at 590 nm according to the instructions of the manufacturer.

### Measurement of TNF-α and IL-1β

BV-2 cells were seeded in 12-well culture plates at a density of 3×10^5^ per well. oAβ_1–42_ was pre-incubated with or without different concentrations of XD4 for 1 h before adding to the cells. The final concentration of oAβ_1–42_ was 5 μM, and the molar ratios of oAβ_1–42_ to XD4 were 1∶2 and 1∶10. After 12 h of incubation, the supernatants were collected to determine the levels of TNF-α and IL-1β released from the activated BV-2 cells using the TNF-α and IL-1β ELISA kits according to the protocols of the manufacturer.

### Statistical Analysis

Data were obtained from at least three separate experiments for each experimental condition. Data were presented as mean ± standard deviation, and their statistical significance was analyzed by one-way ANOVA. Multiple comparisons between the groups were performed using Student–Newman–Keuls (SNK) method.

## Results

### Peptide XD4 Increases Uptake of mAβ_1–42_ in Microglia

**Figure 1:**
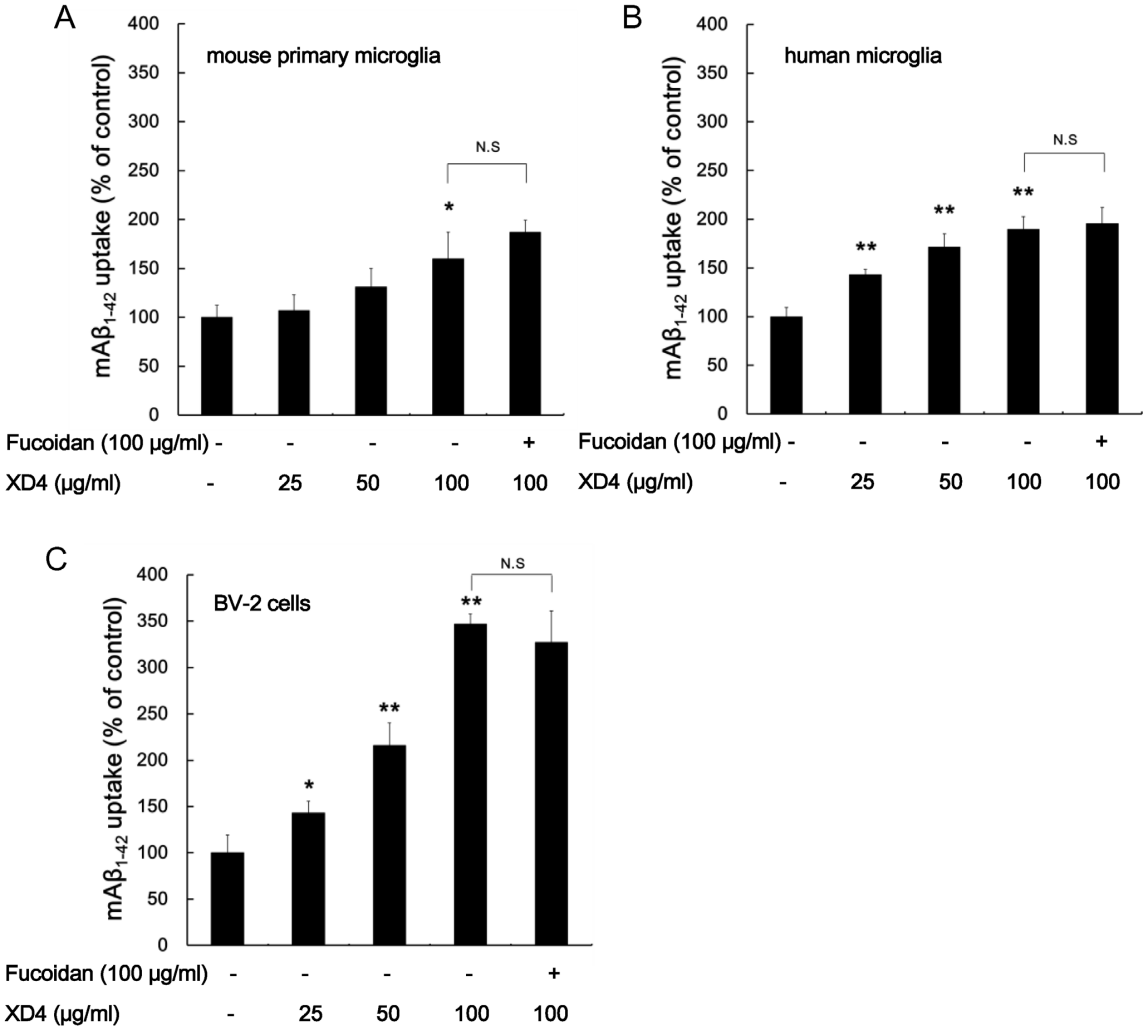
XD4 enhanced Aβ_1–42_ monomer (mAβ_1–42_) uptake in microglia. mAβ_1–42_ was incubated with or without XD4 at the indicated molar ratios for 1 h, and then added to various microglia pretreated with or without fucoidan. After 3 h of incubation, mouse primary microglia (A), human microglia (B), and BV-2 cells (C) were lysed and intracellular mAβ_1–42_ was measured by ELISA. The data showed the uptake value as a percentage of control (compared with mAβ_1–42_ alone, *, p<0.05, **, p<0.01).

Mouse primary microglia, human microglia, and BV-2 cells were treated with mAβ_1–42_ incubated with or without XD4 for 3 h. The intracellular mAβ_1–42_ was measured using immunoassay kits. The results showed that the intracellular Aβ_1–42_ levels were significantly increased in a concentration-dependent manner in all three types of microglial cells with the addition of XD4 (Figure 1). To investigate the mechanism, fucoidan, which is a general ligand and antagonist of scavenger receptors, was added to three types of microglia and incubated for 20 min. Then, mAβ_1–42_ pre-incubated with or without XD4 was added to the cells. The results showed that fucoidan failed to block the mAβ_1–42_ engulfment induced by XD4, suggesting that mAβ_1–42_ may be taken up through a nonsaturable fluid phase macropinocytosis rather than phagocytosis or receptor-mediated endocytosis, which is consistent with a previous report by Mandrekar *et al.* [7]. Moreover, when XD4 and mAβ_1–42_ were added to the cells separately, no increased uptake of mAβ_1–42_ was observed (data not shown), indicating that a complex formed from mAβ_1–42_ and XD4 may be a prerequisite for enhanced internalization of Aβ_1–42_.

### mAβ_1–42_ and XD4 Colocalize in BV-2 Cells

**Figure 2:**
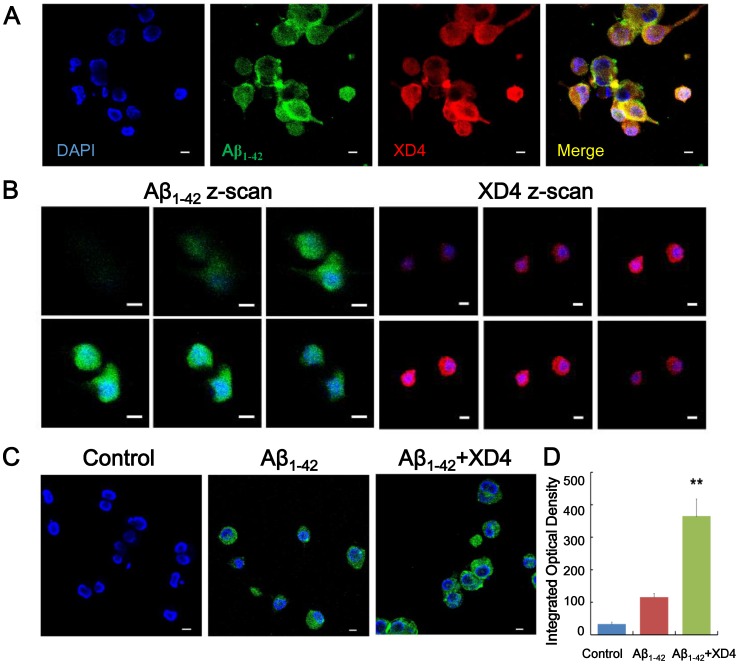
Intracellular localization of mAβ_1–42_ and XD4 in BV-2 cells. Microglia were incubated with mAβ_1–42_ (green) or mAβ_1–42_ pre-incubated with XD4-His-tag (red) for 1.5 h at 37°C and immunostained with 4G8 antibody and anti-His antibody, respectively. Nuclei were stained with DAPI (blue). The cells were visualized by confocal microscope to detect the colocalization of mAβ_1–42_ and XD4 in BV-2 cells (A), internalization of mAβ_1–42_ and XD4 in BV-2 cells by apical-to-distal Z-scan (B), and effect of XD4 on uptake of Aβ_1–42_ (C). Scale bars = 10 μm. (D) The densitometric analysis of 2C with IpWin5 (compared with oAβ_1–42_ alone, **, p<0.01).

To validate the internalization of mAβ_1–42_ and XD4 in the microglia, confocal microscopy was used to visualize the distribution pattern of the two peptides within BV-2 cells. The results showed that internalized mAβ_1–42_ and XD4 colocalized in the cells (Figure 2), further suggesting that mAβ_1–42_ may be internalized as mAβ_1–42_–XD4 complex (Figure 2A). Z-stack confocal microscopy was employed to detect the distribution pattern of mAβ_1–42_ and XD4. Z-stack images, taken at different depths within the cells, indicated that both Aβ_1–42_ and XD4 are located in the cytoplasm, but not in the nuclear region (Figure 2B), which is partly consistent with the previous report that the internalized Aβ_1–42_ is located in the cytoplasm and nuclear region [7]. Moreover, BV-2 cells treated with mAβ_1–42_–XD4 complex displayed a stronger florescence signal of Aβ_1–42_ than those treated by Aβ_1–42_ alone (Figure 2C and 2D), also suggesting that XD4 promoted the uptake of Aβ_1–42_ by microglia.

### Scavenger Receptors are Involved in XD4-mediated oAβ_1–42_ Phagocytosis in Microglia

**Figure 3:**
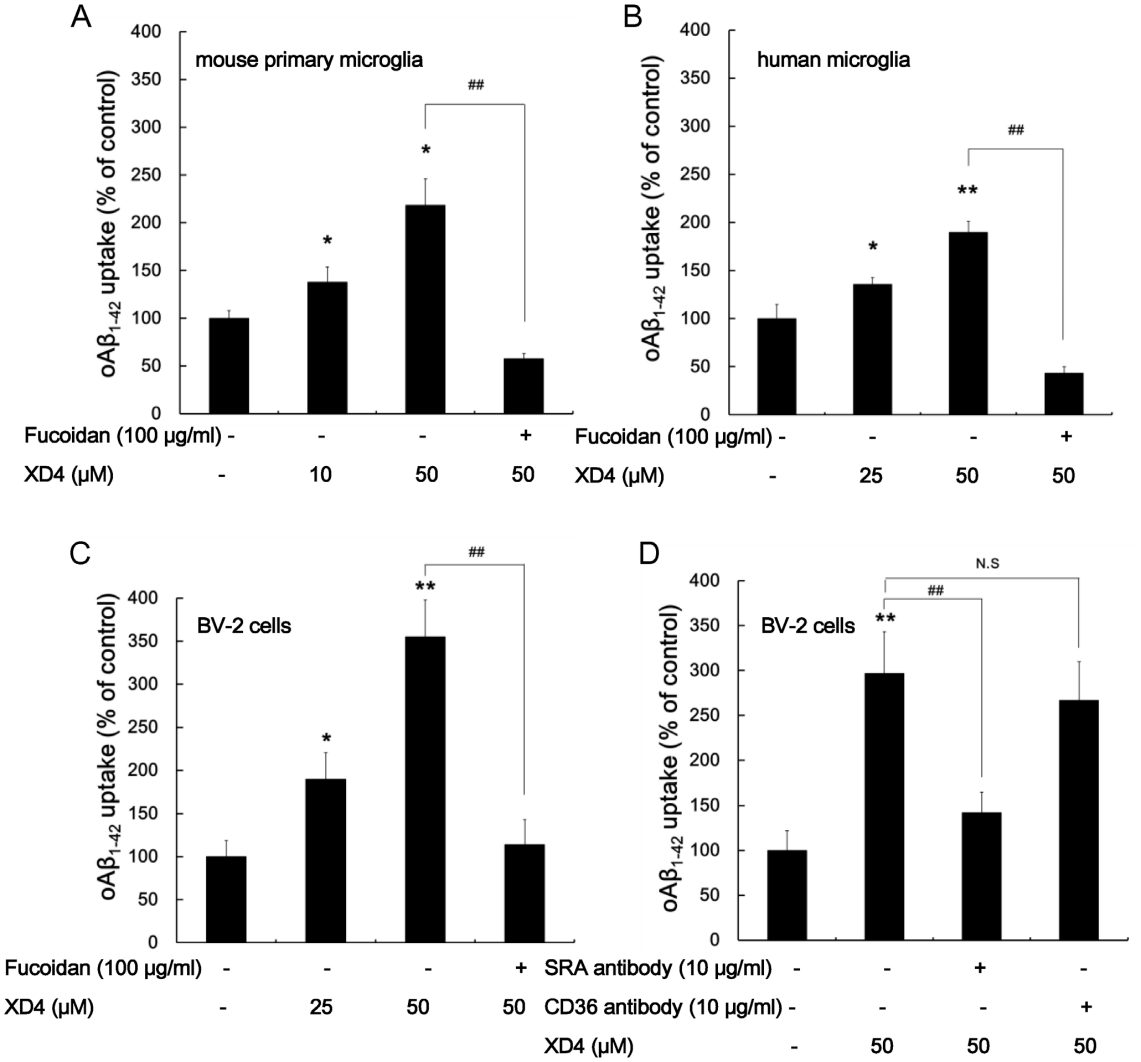
XD4 enhanced oAβ_1–42_ phagocytosis in microglia. oAβ_1–42_ was incubated with or without XD4 for 1 h, and then added to microglia cell lines pretreated with or without fucoidan. After 1.5 h of incubation, mouse primary microglia (A), human microglia (B), and BV-2 cells (C) were lysed, and intracellular oAβ_1–42_ was determined by oligomer-specific ELISA. The data showed the uptake value as a percentage of control. (D) BV-2 cells were incubated with anti-SR-A antibody and anti-CD36 antibody for 30 min, and then exposed to oAβ_1–42_ or oAβ_1–42_-XD4 for 1.5 h. The cells were lysed and the intracellular oAβ_1–42_ was measured by ELISA. All data were standardized as percentage of control (compared with oAβ_1–42_ alone, *, p<0.05, **, p<0.01; compared with oAβ_1–42_+50 μM XD4, ^##^, p<0.01).

To investigate the effect of XD4 on glial internalization of oAβ_1–42_, the intracellular oAβ_1–42_ levels in the three types of microglia in the presence or absence of XD4 were measured. The results showed that XD4 increased oAβ_1–42_ phagocytosis by mouse primary microglia, human microglia, and BV-2 cells in a concentration-dependent manner (Figure 3). However, the uptake of oAβ_1–42_ was drastically hampered by fucoidan in these microglia, suggesting that increased phagocytosis of oAβ_1–42_ induced by XD4 may be mediated by the scavenger receptor, which is consistent with the previous report that internalization of mAβ_1–42_ and oAβ_1–42_ applies distinct mechanisms.

The phagocytosis of Aβ_1–42_ by microglia may be orchestrated by receptor ensembles and dysfunction of any component within the receptor complex [12]. The scavenger receptor-neutralizing antibody attenuates phagocytosis in the microglia and astrocytes [20], [23]. SR-A and CD36 receptors have critical roles in Aβ_1–42_ binding, uptake, and concomitant degradation. To identify which subtype of SRs is involved in XD4-mediated oAβ_1–42_ internalization, BV-2 cells were incubated with the neutralizing antibodies against SR-A or CD36 before the addition of oAβ_1–42_ or oAβ_1–42_-XD4 complex. The results showed that the anti-SR-A antibody reduced the oAβ_1–42_ internalization by 50% in BV-2 cells (Figure 3D), whereas the anti-CD36 antibody did not interfere with oAβ_1–42_ internalization. These data suggest that SR-A receptor has an important role in XD4-mediated oAβ_1–42_ phagocytosis in the microglia.

### XD4 Strengthens the Interaction between SR-A and oAβ_1–42_

**Figure 4:**
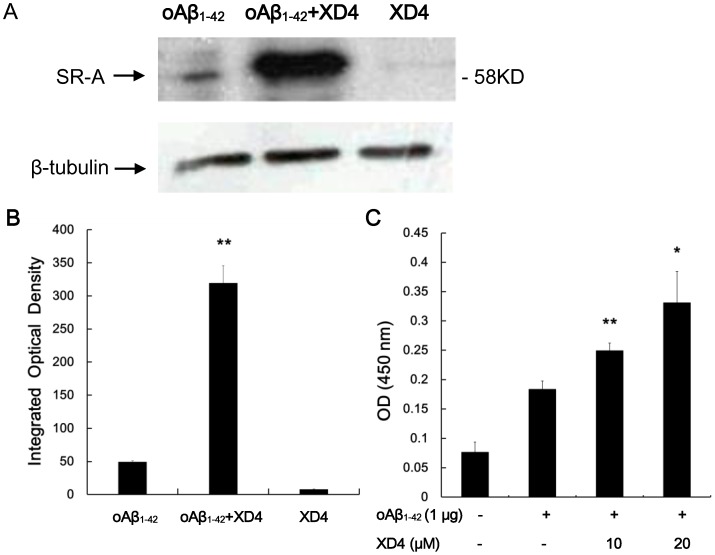
XD4 promoted the interaction between SR-A and oAβ_1–42_. (A) Equal volumes of homogenized membrane fraction of BV-2 cells were added to oAβ_1–42_ or oAβ_1–42_-XD4 preincubated with 6E10-conjugated resin. After 4 h of incubation with gentle agitation, the binding protein from the co-IP process was separated by SDS-PAGE (12% gels) and subjected to immune-blot analysis using anti-SR-A antibody. The control experiment was performed through the same procedure in the absence of oAβ_1–42_. (B) The densitometric analysis was carried out by IpWin5. (C) The affinity of SR-A for oAβ_1–42_ with or without XD4 was tested by ELISA. High binding 96-well ELISA plates were coated with 1 μg oAβ_1–42_ in the presence or absence of XD4 at 4°C overnight. The prepared membrane protein extracts of BV-2 cells were added and then the rat-anti SR-A primary antibody, HRP-conjugated anti-rat secondary antibody was added followed by TMB substrate (compared with oAβ_1–42_ alone, *, p<0.05, **, p<0.01).

To unravel the mechanism through which XD4 increased oAβ_1–42_ phagocytosis in the microglia, the membrane protein fraction of BV-2 cells incubated with oAβ_1–42_ or oAβ_1–42_–XD4 complex was immunoprecipitated using 6E10-coupled resins. The eluted samples were detected by Western blot using anti-SR-A antibody. Consistent with previous report, the samples immunoprecipitated by 6E10 showed the SR-A bands at 58 KD (Figure 4A). When BV-2 cells were incubated with oAβ_1–42_–XD4 complex, the immunoprecipitated samples showed higher amount of SR-A (Figure 4B). Considering that the expression of SR-A protein cannot be induced to express within 2 h during experimental processing, the greater number of SR-A protein observed in the samples with the addition of oAβ_1–42_–XD4 may result from the strengthened interaction of oAβ_1–42_ with SR-A by XD4. This reinforced binding of oAβ_1–42_ to SR-A may increase the phagocytosis of Aβ. In addition, CD36 was not observed in the immunoprecipitated samples, suggesting that XD4 did not affect the interaction of CD36 and Aβ (data not shown).

To measure the affinity of SR-A for oAβ_1–42_ with or without XD4, high binding 96-well ELISA plates were coated with 1 μg oAβ_1–42_ in the presence or absence of XD4 at 4°C overnight. The prepared membrane protein extracts of BV-2 cells were added and then the rat-anti SR-A primary antibody, HRP-conjugated anti-rat secondary antibody was added. The ELISA results indicated that XD4 significantly increased the binding affinity of SR-A for oAβ_1–42_ (Figure 4C), which is consistent with above results.

### XD4 Increases Uptake of mAβ_1–42_ and oAβ_1–42_ in Astrocytes

**Figure 5:**
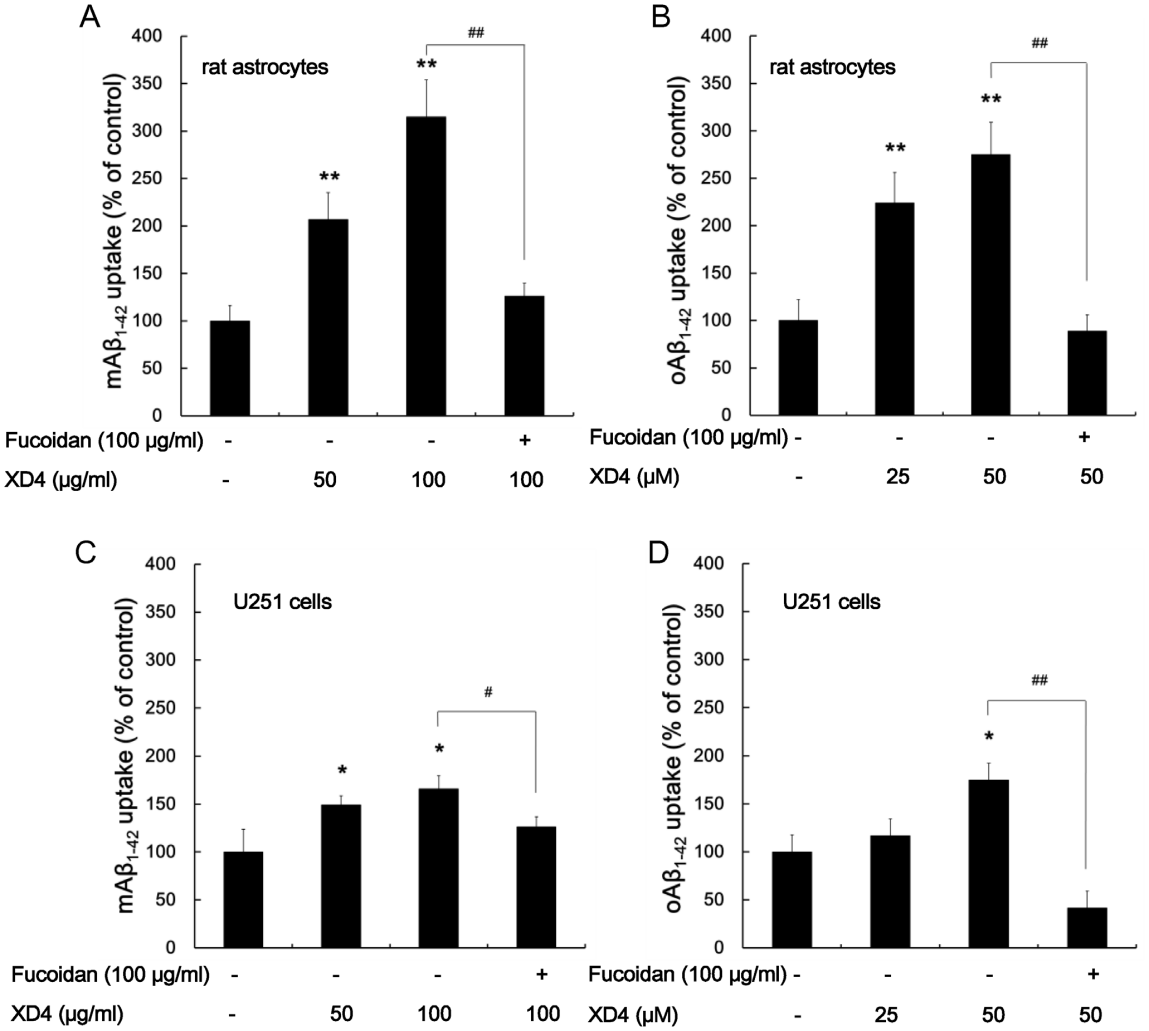
XD4 promoted uptake of Aβ_1–42_ in astrocytes. mAβ_1–42_ or oAβ_1–42_ was incubated with or without XD4 at the indicated molar ratio for 1 h, and then added to astrocyte cells pretreated with or without fucoidan. After 1.5 h of incubation, rat astrocytes (A, B) or U251 cells (C, D) were lysed and intracellular mAβ_1–42_ (A, C) or oAβ_1–42_ (B, D) was determined by ELISA. The data showed the uptake value as a percentage of control (compared with Aβ_1–42_ alone, *, p<0.05, **, p<0.01; compared with Aβ_1–42_+50 μM XD4, ^#^, p<0.05, ^##^, p<0.01).

To test the effect of XD4 on phagocytosis of Aβ_1–42_ in astrocytes, the mAβ_1–42_ and oAβ_1–42_ levels in rat astrocytes and human astroglioma U251 cells pre-incubated with Aβ_1–42_ in the presence or absence of XD4 were detected. Data demonstrated that XD4 significantly increased the uptake of both mAβ_1–42_ and oAβ_1–42_ in rat astrocytes and U251 cells, however, these effects were inhibited by 100 μg/ml fucoidan (Figure 5), indicating that XD4 increased the uptake of mAβ_1–42_ and oAβ_1–42_ through SR, and microglia and astrocytes applied distinct mechanisms to internalize mAβ_1–42_.

### XD4 Attenuates oAβ_1–42_ Cytotoxicity in Glia Cells

**Figure 6:**
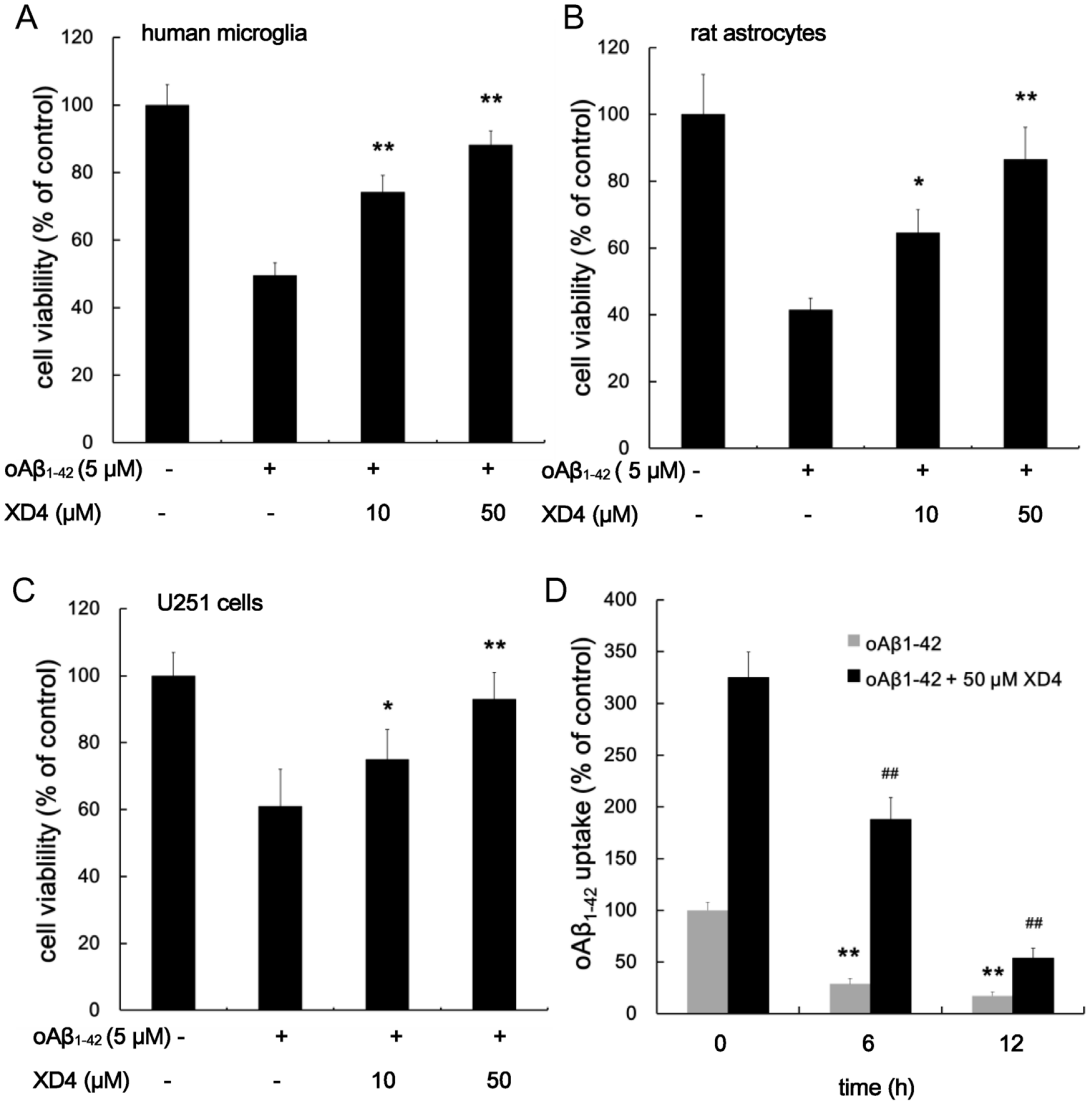
Protective effect of XD4 on oAβ_1-42_-induced cytotoxicity in glia cells. oAβ_1-42_ was pre-incubated with or without different molar ratios of XD4 for 1 h before adding to the human microglia (A) rat astrocytes (B) and U251 cells (C), respectively. After two days of incubation, cell viability was measured using CCK-8 assay. The absorbance of the wells was read at 450 nm using an MD SpectraMax M5 microplate reader. Cell viability was calculated by dividing the absorbance of the wells containing the samples (corrected for background) by the absorbance of wells containing medium alone (corrected for background) (compared with oAβ_1-42_ alone, *, p<0.05, **, p<0.01). (D) The internalized oAβ_1-42_ was cleared in a time-dependent manner. After BV-2 cells were incubated with oAβ_1-42_ for 1.5 h in the presence or absence of 50 μM XD4, the intracellular oAβ_1-42_ levels in BV-2 cells were measured by ELISA at 0 h, 6 h and 12 h, respectively. The data showed the value as a percentage of control (compared with oAβ_1-42_ alone at 0 h, **, p<0.01; compared with oAβ_1-42_+50 μM XD4 at 0 h, ^##^, p<0.01).

Aβ is believed to be a cytotoxic factor that causes neuronal damage in AD progressing. To test whether the enhanced uptake of Aβ_1–42_ induced by XD4 exerts toxic effects on the glia, human microglia, rat astrocytes, and U251 cells were incubated with oAβ_1–42_ in the presence or absence of XD4. The results indicated that the cell viability of all three glial cells incubated with oAβ_1–42_ alone remarkably decreased (Figure 6). However, oAβ_1–42_-induced cytotoxicity can be drastically attenuated by the addition of XD4 in a concentration-dependent manner. Our finding indicated that XD4 can protect glial cells from Aβ_1–42_-induced cytotoxicity, although it obviously enhanced intracellular Aβ levels.

To investigate what happens to the internalized oAβ_1–42_ in the glial cells, the BV-2 cells were incubated with oAβ_1–42_ for 1.5 h in the presence or absence of 50 μM XD4, the intracellular oAβ_1–42_ levels in BV-2 cells were measured by ELISA at 0 h, 6 h and 12 h, respectively. The results showed that the intracellular oAβ_1–42_ levels decreased in a time-dependent manner (Figure 6D). The mechanism of oAβ_1–42_ clearance in microglia may be that the internalized oAβ_1–42_ was trafficked toward lysosomes and degraded by cysteine proteases, including cathepsin B [23].

### oAβ_1–42_-XD4 Complex Activates SR-A

**Figure 7:**
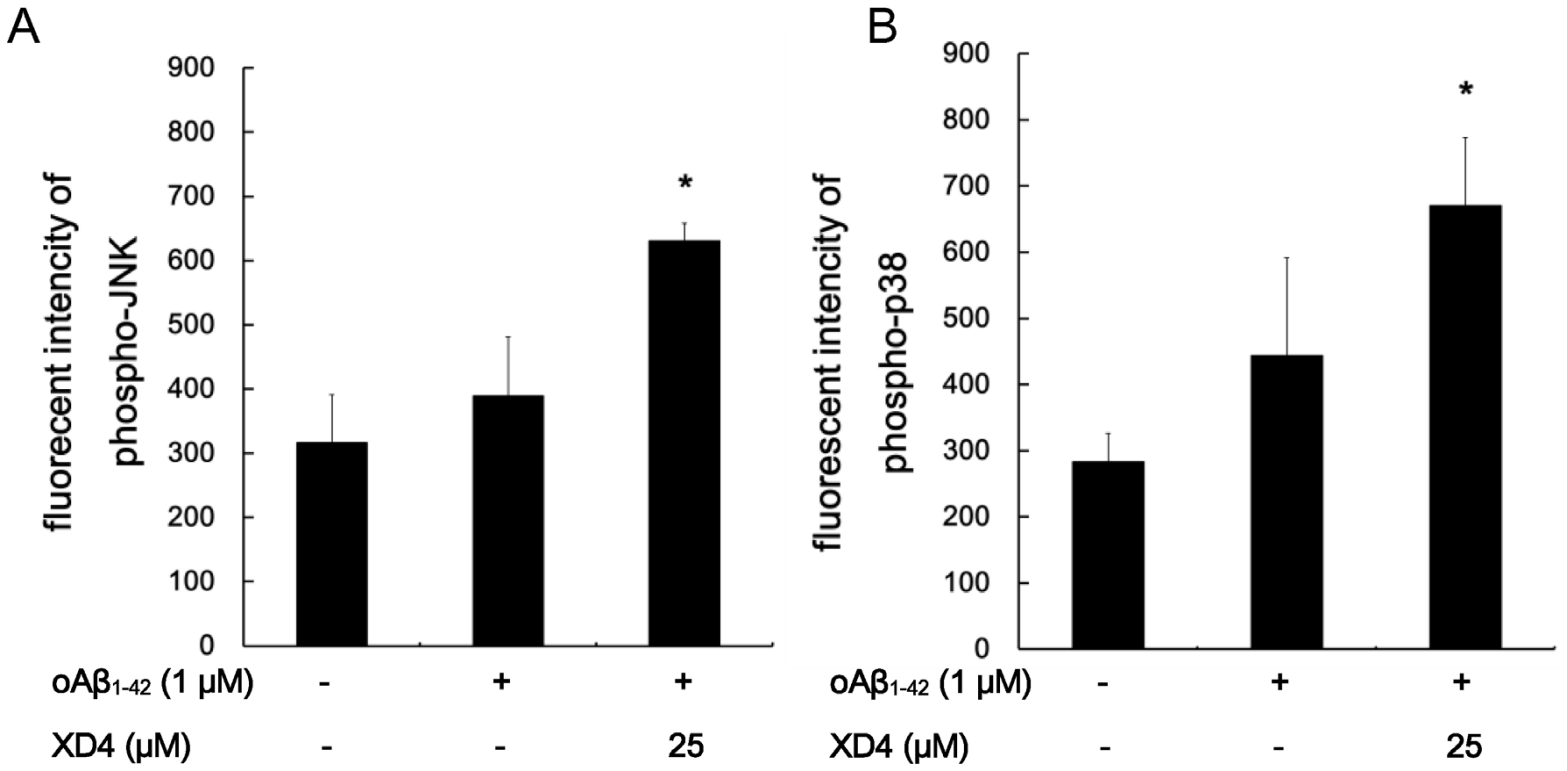
XD4 enhanced JNK and p38 phosphorylation. oAβ_1-42_ or oAβ_1-42_-XD4 was added to the BV-2 cells. After 2 h of incubation, the cells were lysed and the phosphorylated JNK (A) and p38 (B) levels were determined by ELISA (compared with oAβ_1-42_ alone, *, p<0.05).

JNK and p38 MAPK are involved in the SR ligand-regulated signaling cascade in macrophages and glia. The levels of phosphorylated JNK and p38 MAPK were determined to verify the effect of XD4 on oAβ_1–42_ phagocytosis and to explore the mechanism through which XD4 enhances Aβ internalization. The results showed that oAβ_1–42_ treatment did not significantly increase JNK and p38 MAPK phosphorylation in BV-2 cells. However, oAβ_1–42_-XD4 complex enhanced this phosphorylation (Figure 7). These results illustrated that the oAβ_1–42_ - XD4 complex can activate SR-A, thus enhancing the phosphorylation of JNK and p38 MAPK.

### XD4 Reduces oAβ_1–42_-induced Production of TNF-α and IL-1β

**Figure 8:**
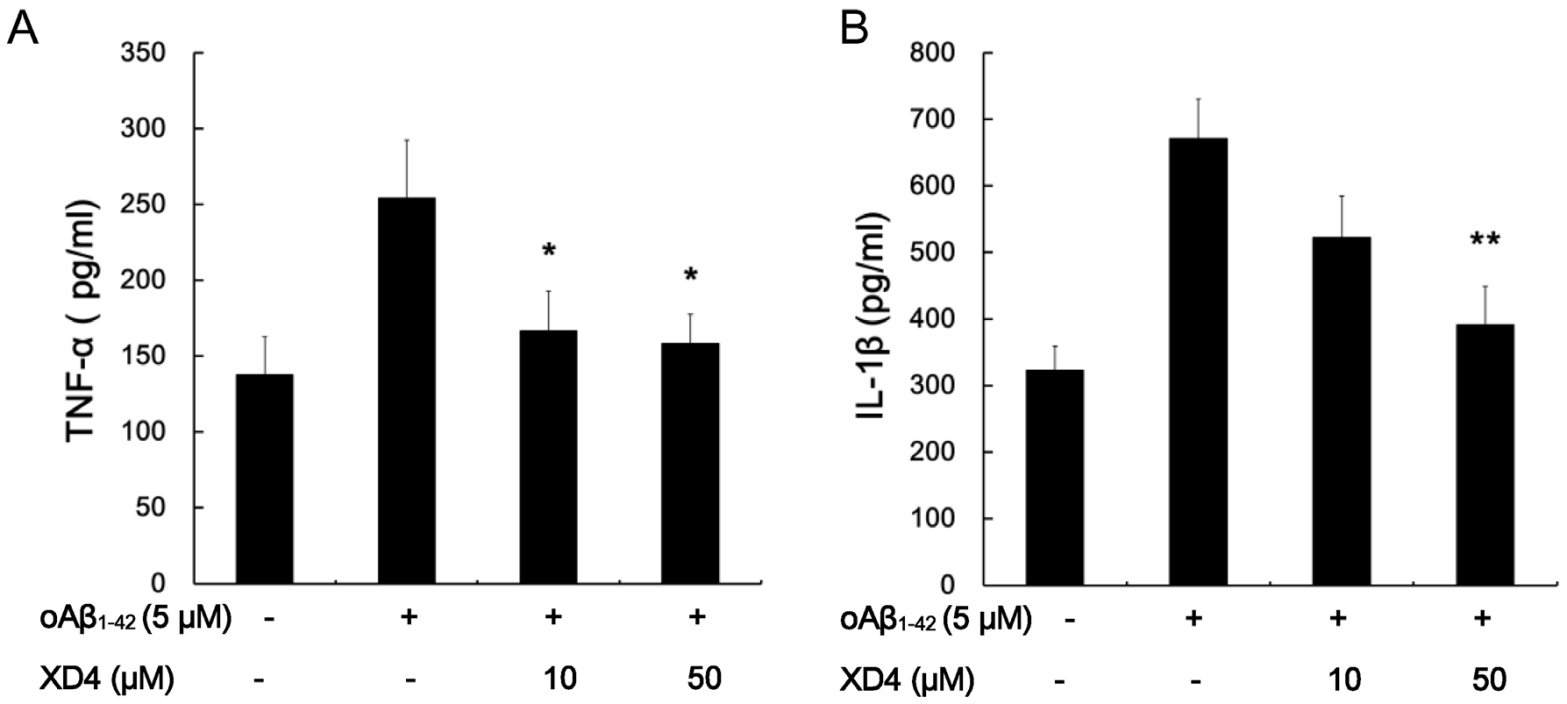
XD4 reduced production of TNF-α and IL-1β. BV-2 cells were incubated with oAβ_1-42_ in the presence or absence of XD4 for 12 h. The supernatants were collected and the levels of TNF-α (A) and IL-1β (B) were determined using ELISA kits (compared with oAβ_1-42_ alone, *, p<0.05, **, p<0.01).

To assess the effect of XD4 on the production of proinflammatory cytokines, BV-2 cells were incubated with oAβ_1–42_ in the presence or absence of XD4 for 12 h. The supernatants were collected for TNF-α and IL-1β measurements by ELISA. Our results showed that oAβ_1–42_ alone significantly increased the generation of TNF-α and IL-1β, whereas XD4 significantly reduced TNF-α and IL-1β levels in the supernatants in a concentration-dependent manner (Figure 8). These findings indicated that XD4 can attenuate oAβ_1–42_-induced proinflammatory cytokine production, although it enhances Aβ uptake.

## Discussion

AD is the most common neurodegenerative dementia with progressive deposition of Aβ within the brain. The soluble or small oligomeric forms of Aβ induce synaptic dysfunction, behavioral deficits, and neuronal degeneration [24]. Therefore, the elimination of toxic Aβ species in the early stages of AD seems to be a promising strategy for rescuing synaptic damage and relieving cognitive impairment [23]. Microglia are major phagocytic cells in the brain. Upon injury, microglia migrate to the lesion sites, recognize the pathogen, and then ramify and mount an immune response in response to the stimulus [25]. The activation and accumulation of microglial cells around Aβ plaques have been reported in the brains of both AD patients and mouse models [26]. Microglia can engulf Aβ and degrade Aβ, but this action is a dual sword. Although microglia exert a protective role, possibly by internalization and degradation of Aβ at initial stages of AD, they may also lose Aβ phagocytosis and induce neuronal damage by releasing pro-inflammatory and toxic agents, especially at the late stages of AD. Therefore, turning the deleterious state of microglia to beneficial state is a reasonable strategy for AD treatment. To the best of our knowledge, no agent that promotes Aβ clearance by SR-A in microglia has been reported. In this study, we showed that glia phagocytosis can be enhanced by peptide XD4.

Aside from microglia, astrocytes are also considered main phagocytes that can engulf Aβ [20]. Glia can engulf Aβ in several ways, such as nonsaturable, fluid phase macropinocytosis and phagocytosis [7], [11], [23]. Our results demonstrated that XD4 increased the uptake of Aβ monomers by microglia and astrocytes, however, this effect can be blocked by fucoidan in astrocytes rather than in microglia, suggesting that different glial cells apply different mechanisms to engulf Aβ monomers. Microglia engulf mAβ_1–42_ through nonsaturable, fluid phase macropinocytosis, whereas astrocytes engulf mAβ_1–42_ through phagocytosis by SR-A, which consistent with previous reports [19], [27].

Previous reports indicated that SR was also involved in phagocytosis of fibrillar Aβ by microglia [11], [28]. Our present results showed that SR-A was involved in the uptake of Aβ oligomers by microglia, and XD4 enhanced the phagocytosis of Aβ (Figure 3), which was confirmed by the blockage of phagocytosis with fucoidan and anti-SR-A antibody. Moreover, the phagocytosis of Aβ oligomers by astrocytes was also enhanced by XD4, and this enhanced phagocytosis was significantly decreased by fucoidan, indicating that XD4 enhanced the internalization of oAβ_1–42_ in astrocytes also via the SR-A pathway. Our immunoprecipation and Western blot results suggested that the enhanced binding of oAβ_1–42_ to SR-A by XD4 contributed to the enhanced phagocytosis of oAβ_1–42_.

Our previous study showed that XD4 did not inhibit Aβ aggregation, but attenuated Aβ_1–42_-induced cytotoxicity in SH-SY5Y cells [29]. In this study, we showed that XD4 also inhibited oAβ_1–42_-induced cytotoxicity in glial cells and presented a neuroprotective effect, although more oAβ_1–42_ was internalized into the cells, which may be attributed to the fact that the interaction between XD4 and Aβ aggregates forms a harmless structure of Aβ. The activation of SRs by the binding of ligand such as fucoidan can trigger downstream signal transduction pathways in glial cells, increasing the phosphorylation of diverse kinase, such as JNK and p38 [17], [30]. Our finding indicated that oAβ_1–42_-XD4 complex can significantly increase the phosphorylation of JNK and p38 MAPK, further confirming that SR-A is involved in XD4-mediated Aβ phagocytosis.

Increasing evidence has shown high levels of inflammatory mediators in the brain of AD patients. Activated microglia cells by Aβ accumulation release high levels of proinflammatory cytokines, such as TNF-α and IL-1β, which may further cause neuronal dysfunction [31], [32]. Although XD4 activated SR-A, it did not increase the generation of TNF-α and IL-1β in BV-2 cells induced by oAβ_1–42_ in vitro and in vivo [28]. This phenomenon may be attributed to the fact that the enhanced phagocytosis lowered the levels of extracellular Aβ and reduced the Aβ-mediated stimulation to the cells, decreasing the release of TNF-α and IL-1β. Moreover, previous reports have demonstrated that the binding of ligands to SR-A attenuated the TLR4-mediated inflammation responses [33], which may partly explain the reduced production of proinflammatory cytokines induced by XD4.

Therefore, XD4 enhanced the glial uptake of Aβ monomers and oligomers through phagocytosis or macropinocytosis by strengthening the binding of Aβ to SR-A, inhibited oAβ_1–42_-induced cytotoxicity, and reduced the production of proinflammatory cytokines, including TNF-α and IL-1β. As a short peptide, XD4 may have lower immunogenicity than antibodies, and is suitable for further engineering to improve specificity and stability. In conjunction with our previous findings that XD4 attenuated memory deficits in AD transgenic mice and reduced amyloid plaque burden and Aβ 40/42 levels, XD4 may be a promising therapeutic agent for AD treatment.
